# Adherence with statins in a real-life setting is better when associated cardiovascular risk factors increase: a cohort study

**DOI:** 10.1186/1471-2261-11-46

**Published:** 2011-07-26

**Authors:** Philippe Latry, Mathieu Molimard, Bernard Dedieu, Thierry Couffinhal, Bernard Bégaud, Karin Martin-Latry

**Affiliations:** 1 Direction Régionale du Service Médical de l'Assurance Maladie d'Aquitaine, Cnam-TS, Bordeaux, (33000), France; 2 Univ. de Bordeaux, Bordeaux, (33000), France; 3 U 657, INSERM, Bordeaux, (33000), France; 4 Centre d'exploration, de prévention et de traitement de l'athérosclérose, hôpital Haut-Lévêque, Pessac, (33600), France; 5 U 1034, INSERM, Pessac, (33600), France

**Keywords:** Hydroxymethylglutaryl-CoA Reductase Inhibitors, Risk factors, Medication adherence, Databases, Factual, Pharmacoepidemiology, Insurance, Health, Reimbursement

## Abstract

**Background:**

While the factors for poor adherence for treatment with statins have been highlighted, the impact of their combination on adherence is not clear.

**Aims:**

To estimate adherence for statins and whether it differs according to the number of cardiovascular risk factors.

**Methods:**

A cohort study was conducted using data from the main French national health insurance system reimbursement database. Newly treated patients with statins between September 1 and December 31, 2004 were included. Patients were followed up 15 months. The cohort was split into three groups according to their number of additional cardiovascular risk factors that included age and gender, diabetes mellitus and cardiovascular disease (using co-medications as a *proxy*). Adherence was assessed for each group by using four parameters: *(i) *proportion of days covered by statins, *(ii) *regularity of the treatment over time, *(iii) *persistence, and *(iv) *the refill delay.

**Results:**

16,397 newly treated patients were identified. Of these statin users, 21.7% did not have additional cardiovascular risk factors. Thirty-one percent had two cardiovascular risk factors and 47% had at least three risk factors. All the parameters showed a sub-optimal adherence whatever the group: days covered ranged from 56% to 72%, regularity ranged from 23% to 33% and persistence ranged from 44% to 59%, but adherence was better for those with a higher number of cardiovascular risk factors.

**Conclusions:**

The results confirm that long-term drug treatments are a difficult challenge, particularly in patients at lower risk and invite to the development of therapeutic education.

## Background

Coronary Heart Disease (CHD) remains a major cause of mortality and morbidity in developed countries and dyslipidemia is one of the risk factors for which drugs have been marketed. Statins are by far the principal class used for hypercholesterolemia and their efficacy in reducing the occurrence of cardiovascular adverse clinical outcomes has been clearly documented during the last two decades [1-11]. The impact of this class on the whole drug reimbursement cost has regularly and dramatically increased during recent years. For example in France in 2007, reimbursement of statins accounted for about 800,000 million euros. In this context, the optimized use and public health impact of this class becomes a key issue for health policy with regards to the effectiveness of a drug and its direct and indirect cost. However, several studies have clearly shown that the characteristics of patients and treatment patterns may differ from those of randomized clinical trials: e.g., age, gender, dosage and duration of treatment [12-14]. Moreover, several studies have shown poor adherence to statin treatments and several associated factors have been highlighted: younger age, insufficient revenue, absence of cardiovascular morbidity, women, number of coprescribed drugs [15-24]. Thus, it is essential to identify all the risk factors and their impact in order to act on them if possible. While the factors for poor adherence have been highlighted, the impact of their combination on adherence is not clear. The aim of this study was to estimate whether adherence differs according to the number of cardiovascular risk factors.

## Methods

### Sources of data

This cohort study was performed using anonymous data from the main French health insurance system: the *Caisse Nationale d'Assurance Maladie des Travailleurs Salariés (Cnam-TS) *database of the Aquitaine region of southwest France. This database concerned 2.5 million patients. The Cnam-TS refunds patients 65% of the cost of statin treatment, whatever the type of drugs or their indication. There is no limitation on the amount of drugs to be reimbursed. The 35% of the cost are refunded by a private health insurance. Having such private insurance is very common in France. The refund rate is 100% for patients with insufficient income and for patients suffering from permanent serious disease such as myocardial infarction. This database has been previously described [25]. Data extracted from this database were demographic characteristics of the users, prescribers' specialty, the name of drugs submitted for reimbursement and vital status. Conversely, there is no data regarding diagnosis, daily dose, results of laboratory data or details regarding stays in public hospitals.

### Population

Patients were included if they submitted a reimbursement form for a prescription for statins between September 1 and December 31, 2004, and did not receive any statin treatment for 6 months previous to this. Index date was the date of the first reimbursement claim for a statin in the database during inclusion period. There were no exclusion criteria and patients were followed-up for 15 months after index date.

The new users were split into three groups according to their number of cardiovascular risk factors that included age and co-morbidities. In the database, in addition to statin treatment used as a *proxy *for the risk factor hypercholesterolemia, three other risk factors were identified: age over 50 years for men and 60 for women, diabetes mellitus, and other cardiovascular disease (CVD). As the French reimbursement databases do not include medical data, Diabetes mellitus and CVD were identified using treatments reimbursed in this indication as a *proxy*: insulin and oral hypoglycemiants for diabetes mellitus and central antihypertensives, beta-blockers, diuretics, calcium channel blockers, ACE inhibitors, angiotensin II receptor antagonists for CVD.

Thus, three groups were defined:

1 risk factor: patients with hypercholesterolemia (statin) aged under 50 years for men and 60 years for women.

2 risk factors: patients with hypercholesterolemia (statin) with one of the following: aged over 50 years for men and 60 years for women, diabetes or CVD co-morbidity (the latter were defined using reimbursement of drugs concomitantly with the statin index date, within a two-month interval).

At least 3 risk factors: patients with hypercholesterolemia (statin) with two or more of the following: aged over 50 years for men and 60 years for women, diabetes or CVD co-morbidity.

Prescriber specialty, patient demographic data, vital status, statins between the September 1, 2004 and June 30, 2006 were collected.

### Drug exposure

All drugs were identified in the database according to the Anatomical Therapeutic Chemical (ATC) classification system of the World Health Organization [26]. In France, all the statins are delivered in non-devisable units that most-often contain supply for 28 days of treatment.

### Assessment of adherence

Assessing adherence to drug treatments is questionable if a single criterion is used [27-29]. Therefore, we considered four criteria: *(i) *proportion of days covered by statins (reimbursement of the appropriate quantity of medication for the considered period of time), (*ii*) regularity of the treatment over time, (*iii*) persistence of treatment, and (*iv*) refill delay.

The proportion of days covered during the study period (also termed medication availability) was estimated using the "Continuous Multiple-interval measures of medication Availability" (CMA) definition [30-33]. The CMA is defined as the sum of the days' supply of medication divided by the number of days between the first fill and the last refill. The theoretical days' supply is calculated by dividing the number of units dispensed by the daily dose for the drug considered, here the studied statin. The daily dose is the recommended dose per day for its main indication in adults. The CMA was assessed both using a cut-off at 80% (a CMA lower than 80% was considered as unsatisfactory in several previous studies) and as a continuous variable to characterize the distribution of the CMA in the study population.

The regularity of treatment was assessed by computing the mean of the Continuous Multiple-interval measures of medication Gaps (CMG) [31,33], defined as the ratio between the sum of days without treatment (without a day's supply) and the number of days between the first and the last prescription reimbursed. Therefore, CMG is 1 *i.e*. 100% if there was no gap, *i.e*. no drug without treatment during the period. CMA and CMG are restricted to patients with a refill.

The treatment persistence was the percentage of patients still treated at the end of the period and described by a Kaplan Meier survival curve analysis [34]. Unlike the two preceding measures, analyses were performed on all patients, even those with only one reimbursement. Discontinuation was defined as a minimum gap of 90 days between the theoretical end date of prescription reimbursed (based on a days' supply) and the starting date of the next one. A switch between statins was not considered as a treatment discontinuation.

The refill delay was assessed by using the delay between the first two reimbursements. A short delay was assumed to reflect a good understanding of the chronic character of the pathology requiring regular and long-term treatment. Conversely, a long renewal delay could reflect the patient's misunderstanding of his pathology and its management. It could also signal the occurrence of an adverse effect. The data contained in the reimbursement database do not allow these two possibilities to be differentiated.

Follow-up was censored if exceeding the end of the study period (June 30, 2006), or if a patient died or moved out of the Aquitaine region.

### Ethics/consent

We performed an observational study on anonymous data. Thus, considering the French legislation, it does not need to be approved by an ethic committee.

### Statistical analysis

Analysis was performed using SAS software version 9.1 for PC; the Student t and Chi tests were used. Kaplan-Meier analysis was used to estimate persistence rate since it takes into account censored observations.

## Results

### Patients and treatment characteristics

16,397 newly-treated patients were included for the study. Mean age was 61 years and the proportion of men was 48%. The most delivered drugs were pravastatin (36.3%) and atorvastatin (32.3%) (Table 1). The percentage of switches to another statin during the study period ranged from 1.8% for patients initially treated with atorvastatin to 3.3% for patients initially treated with rosuvastatin. Rosuvastatin was the drug towards which the proportion of switches was the highest (8.4 *versus *1.3 to 2.7% for other drugs). General practitioners were by far the most prevalent prescribers (67.1%) before cardiologists (4.8%) in second place.

**Table 1:** Newly treated patients and treatment characteristics

	n = 16,397	
**Patients n, (%)**		
**Male**	7,827	(47.7)
**Female**	8,480	(51.8)
**Not available**	90	(0.5)
**Median age (male) +/-SD**	59 years	13.5
**Median age (female) +/- SD**	63 years	13.8
**Initial statin treatment, n (%)**		
**Atorvastatin**	5,296	(32.3)
**Fluvastatin**	1,775	(10.8)
**Pravastatin**	5,961	(36.3)
**Rosuvastatin**	791	(4.8)
**Simvastatin**	2,589	(15.8)
**Groups, n (%)**		
**One risk factor (statin alone)**	3,560	(21.7)
**Two risk factors**	5,072	(30.9)
**At least three risk factors**	7,765	(47.4)

Stratified by cardiovascular risk (CV), 21.7% had one risk factor (hypercholesterolemia but younger age), 30.9% had two risk factors (hypercholesterolemia with one of either advanced age or co-morbidity) and 47.4% three or more risk factors (hypercholesterolemia with two or more of either advanced age or co-morbity, Table 1). Patient characteristics were quite different across CV risk groups: the mean age and proportion of men significantly increased by number of associated risk factor (p < 10^-5^) and as the percentage of the males also significantly increased (p < 10^-3^) (Table 2). The percentage of deaths during follow-up was 5- to 10-fold higher in the group with statin and at least two other risk factors and the difference was statistically significant, while delay of occurrence of the death seemed to be identical (Table 2).

**Table 2:** characteristics of the study population with statin according to the group

	One risk factor (statin alone) n = 3,560		Two risk factors* n = 5,072		At least three risk factors* n = 7,765		Total n = 16,397	
**Demographic characteristics**								
Male (%)	1,139	(37.6)	2,374	(46.8)	4,097	(52.8)	7,810	(47.6)
**Age**						(10.1)	31.2	(9.5)
Mean (sd)	45.3	(9.7)	58.8	(10.9)	69.0			
Median	46		59		69.0			
Extremes	2-59		2-95		18-102			
**Vital status**								
Number of deaths during the study period (%)	13 (0.37)		34 (0.67)		294 (3.79)			
Mean delay of occurrence (death), months	310		284		283			
(Min-max delay, days)	(188-433)		(224-345)		(262-304)			

*: other risk factors include age, diabetes mellitus and cardiovascular co-morbidities.

### Medication adherence in new users

#### Medication availability

The CMA was better for patients with increasing CV risk. CMA at 15 months varied from an average of 56% (67% with a CMA ≤ 80%) for those with one risk factor to 72% (43.3% with a CMA ≤ 80%) for patients with three or more risk factors (Table 3) and the differences were statistically significant.

**Table 3:** adherence to statin treatment for new users

Type of indicator	Parameters	One risk factor (statin alone)	Two risk factors*	At least three risk factors*
	**n**=	3,560	**5,072**	**7,765**
**Medication availability**	**CMA, mean % **[CI**_95_**]	56 [54.7;57.2]	64.4 [63.4;65.5]	72.2 [71.9;73.6]
	**CMA < 80%**, n (%)	2,378 (67.0)	2,889 (57.0)	3,361 (43.3)
**Persistence**	**Cumulative rate for persistence** **at 15 months **% [CI**_95_**]	44.3 [42.7;45.9]	50.1 [48.7;51.5]	59.4 [58.3;60.5]
**Regularity**	**CMG**, mean % [CI**_95_**]	33.4 [32.5;34.3]	29.2 [28.5;29.9]	23.2 [22.7;23.8]
**Refill delay**	**mean **[CI**_95_**], days	31.4 [31.0;31.8]	30.2 [29.9;30.5]	28.6 [28.3;28.8]

CMA: Continuous Multiple-interval measures of medication Availability; CMG: Continuous Multiple-interval measures of medication Gaps.

*: other risk factors include age, diabetes mellitus and cardiovascular co-morbidities

#### Regularity

Regularity was better for patients with three or more risk factors, even if almost one third of the study period was not covered with the treatment, whatever the group (Table 3) (p < 10^-3^).

#### Persistence

**Figure 1:**
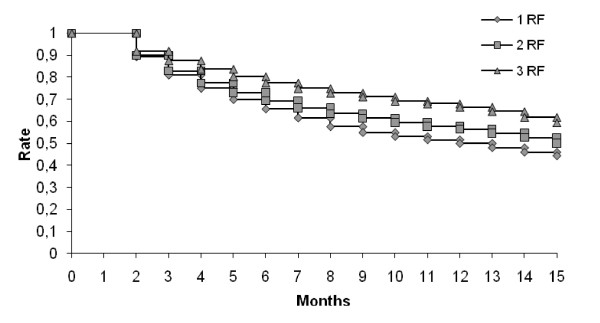
**Persistence with statin treatment according to number of risk factors**. (One risk factor: hypercholesterolemia (statin) and aged under 50 years for men and 60 years for women, Two risk factors: hypercholesterolemia (statin) with one other cardiovascular risk factor (age or co-morbidity), Three risk factors: statin with at least two other cardiovascular risk factors.

Persistence rates varied according to groups, and was significantly worst in those with one risk factor, 44.3% still being treated at 15 months compared to 50.1% for those with two risk factors, and 59.4% of those with three risk factors (Table 3; Figure 1) (p < 10^-3^).

#### Refill delay

Despite the variability of times to first renewal, for the majority of patients (68%) this was one month.

## Discussion

To our knowledge, this study is the first conducted in France using a reimbursement database and a large population to assess patterns and adherence to statin treatment. In this country, reimbursement expenditure for this therapeutic class represented 6% of all drug reimbursements in 2007, making assessment of rationale meaningful. It is also one of the first to explore the associated CV risk factors, including age and co-morbid conditions. The results show an overall poor adherence to treatment in new users with all the parameters explored. However, adherence increased with the number of associated risk factors. A working hypothesis is that patients with several risks are probably more aware of the risk and more confident in their treatment. This is supported by the Health Belief Model that predicts a person will take a health-related action if he/she can avoid a negative health condition [35].

This is an interesting and innovative approach as the impact of each risk factor is usually assessed individually or adherence is compared with regard to the level of prevention (primary *versus *secondary) [19,22,36].

As adherence is a multifaceted question, several standardized parameters were used. These have been extensively discussed in the literature [28,31]. Several remarks can be made for each parameter studied. Concerning the availability of medication (CMA), the poor results observed are in accordance with other studies that have assessed the CMA [16,23]. One factor that may be debated is the use of 80% treatment coverage this parameter as the cut-off for assessing adherence. This is commonly used in the literature, yet it may not be appropriate for all types of drugs [37]. For this reason, we described CMA both as a categorical (≤ 80%) and continuous variable (mean CMA with 95% CI). Even so, the conclusions remain unchanged. Another point is that if patients with more risk factors are on higher doses, their theoretical days supply will appear higher and adherence will appear better. However, in France the statin dose is the same whatever the number of risk factors is the same whatever the number of risk factors, the dose only is changing.

Although not necessarily the case, The CMG values in this study mirrored the CMA values for each group. The proportion of persistence at the end of the 15-month follow-up, *i.e*. 44 to 59%, is comparable to other findings for statins in the literature: 33% at the end of the first year [38], 47 to 83% at the end of the second [39-41], 45 to 61% after three years [18,22] and 52 to 83% at five years [42]. Variations across studies appear to ensue mainly from the population considered, duration of follow-up and definition of discontinuation. In the current study, discontinuation was defined as the absence of any filed statin reimbursement during at least 90 consecutive days. This period was chosen as a conservative approach and takes into account the mode of drug dispensation in France that is often for one month's supply. Consideration of a shorter period would have led to even worse adherence (data not shown).

The CMA results were in accordance with those for persistence: patients with a CMA ≤ 80% were less persistent, whatever their groups (data not shown). Therefore, patients who discontinued treatment were likely the same as those who did not take their medications regularly, and vice versa. However, even if adherence was poor, the patients seemed to have a good understanding of the value of their treatment, as the delay between the first and second delivery was as expected, *i.e*. one month.

The mean refill delay is slightly higher than expected if we consider the 28-non-devisable units of the statin's boxes. Some explanations for this result are lack of understanding, occurrence of an adverse event, inability to get to a pharmacy on time or forgetfulness.

Focusing on treatment patterns, the high percentage of pravastatin could in part be explained by the fact that the national health insurance system puts pressure on physicians to prescribe the cheapest drug of a given class. The proportion of switches was similar across molecules except for rosuvastatin, which had the highest rate of switch towards it. This might reflect the indication of this drug which is prescribed in France, as a second-line therapy.

The poor adherence observed in our study may ensue from several parameters involving prescribers, patients or both: lack of confidence between patients and doctors; insufficient explanation about treatment value and use, *a fortiori *with such a drug that has no immediate clinical consequences in case of non-adherence; underestimation or denial of the seriousness of the disease [43]. There are also the adverse effects of statins, mainly myalgia which if serious, could lead to discontinuation.

The study has several strengths. For instance, the large number of subjects allowed the comparison of three groups and the use of a conservative definition of discontinuation. Furthermore, a database study is not likely to alter the behavior of either prescribers or patients.

However, a study conducted using a reimbursement database suffers from some limitations. Patients may have been classified as having discontinued if these move outside the administrative region (Aquitaine) but this can be considered as only marginal as the population over 40 years of age are geographically quite stable. Discontinuation for financial reasons is also improbable owing to the extent of the national health insurance system, which covers underprivileged and unemployed people. Conversely, the cost of statins is high enough to practically preclude claims for reimbursement not to be made (mean price: 30 euros, *i.e*. 40 USD, 28 GBP). The reimbursement database used here does not include medical data such as indication or previous medical history and therefore a *proxy *was used to define the presence of diabetes mellitus and CVD. In the USA this is less of an issue, but health insurance databases cross-linked to medical databases in European countries remain exceptions, such as the MEMO in Tayside, Scotland [44]. The use of such proxies may be considered as the greatest limitation of reimbursement database for this study. However for statins, hypercholesterolemia is the only indication for this drug, although lipid testing results were not available in the database. With regards to CVD co-morbidity, the indications of the drugs used as proxies were extensive and some may be used for diseases other than CVD (mainly beta-blockers). This may have led to a minimal number of misclassifications for those with CVD co-morbity. The event that led patients to be treated for type II prevention (not investigated here) may not have been captured by the proxies for CV risk. If one considers that such events are themselves risk factors for subsequent events, then these patients may have been miss-classified as patients with fewer associated risk factors. Taken together with the conservative minimal 90 day period considered for discontinuation, the results presented are likely to be an underestimation of adherence. In this study, we cannot explore the extent to which the findings are affected by adjustment for reimbursement level as in France the patients do not pay for their statin' treatments.

At last one must be kept in mind that, as previously demonstrated [45], the adherence will be probably better for patients with past prescriptions for medications for chronic conditions.

## Conclusions

Overall, adherence to statins was poor, but better for those with a higher number of associated CV risk factors. The results confirm that long-term drug treatments are a difficult challenge, particularly for patients who may not see the benefit or feel that they are at risk.

This study is a strong base for the promotion of therapeutic education in the cardiovascular field.

## List of abbreviations

ATC: Anatomical Therapeutic Chemical; CVD: Cardiovascular disease; CV: Cardiovascular risk; CMA: Continuous Multiple-interval measures of medication Availability"; CMG: Continuous Multiple-interval measures of medication Gaps; CHD: Coronary Heart Disease.

## Competing interests

The authors declare that they have no competing interests.

## Authors' contributions

KML, PhL, BB, TC and MM designed the study. BD coordinated data collection. PhL, KML performed analyses. KML, PhL, BB and MM interpreted the results. KML, PhL, BB, TC and MM contributed to writing the paper. KML is the guarantor for the paper. All authors read and approved the final manuscript.

## Pre-publication history

The pre-publication history for this paper can be accessed here:

http://www.biomedcentral.com/1471-2261/11/46/prepub
